# Soil Diversity as Affected by Land Use in China: Consequences for Soil Protection

**DOI:** 10.1155/2014/913852

**Published:** 2014-08-27

**Authors:** Wei Shangguan, Peng Gong, Lu Liang, YongJiu Dai, Keli Zhang

**Affiliations:** ^1^State Key Laboratory of Earth Surface Processes and Resource Ecology, College of Global Change and Earth System Science, Beijing Normal University, Beijing 100875, China; ^2^Ministry of Education Key Laboratory for Earth System Modeling, Center for Earth System Science, Tsinghua University, Beijing 100084, China; ^3^Department of Environmental Science, Policy and Management, University of California, Berkeley, CA 94720, USA; ^4^Joint Center for Global Change Studies, Beijing 100875, China; ^5^School of Geography, Beijing Normal University, Beijing 100875, China

## Abstract

Rapid land-use change in recent decades in China and its impact on terrestrial biodiversity have been widely studied, particularly at local and regional scales. However, the effect of land-use change on the diversity of soils that support the terrestrial biological system has rarely been studied. Here, we report the first effort to assess the impact of land-use change on soil diversity for the entire nation of China. Soil diversity and land-use effects were analyzed spatially in grids and provinces. The land-use effects on different soils were uneven. Anthropogenic soils occupied approximately 12% of the total soil area, which had already replaced the original natural soils. About 7.5% of the natural soil classes in China were in danger of substantial loss, due to the disturbance of agriculture and construction. More than 80% of the endangered soils were unprotected due to the overlook of soil diversity. The protection of soil diversity should be integrated into future conservation activities.

## 1. Introduction

As human impacts on environment reach farther and deeper into relatively undisturbed areas of the world, we begin to protect or conserve various components of the earth that we have valued from ancient times [1]. In the last several decades, the concern over the fate of biological diversity has led to more efforts devoted to the monitoring and protection of the complete variety of genes, species, and ecosystems [2]. It has been widely recognized that one of the major consequences of land use is the loss of biodiversity [3]. However, soils, as the foundation of the terrestrial ecosystems, are rarely given careful consideration in the development of biodiversity and geodiversity planning. Amundson et al. [4] conducted the first comprehensive assessment of the human impact on soil diversity in the USA by defining four types of rare or uncommon soils. Papa et al. [5] found that land-use change was a cause of loss of pedodiversity in Sicily. The possible use of the concept of pedodiversity to select and delineate natural soil reserves has been discussed, and the damage of urbanization to top quality soils has been widely recognized [6–10]. China, as the most populated country in the world, has experienced tremendous land-use change in the past few decades. The pressure on soil resources in China has been widely recognized [11, 12]. However, a comprehensive study on the effect of land use on soil diversity in rapidly developing countries, such as China, does not exist. Such a study is urgently needed before rare natural soil heritages are ruined without even being noticed.

Here, we investigated the abundance and rarity of soils at the national scale of China. Then, we assessed land-use effects on soil diversity and mapped the distribution of endangered soils in China due to agricultural expansion and urbanization. Finally, we assessed the extent of protection of endangered soils by nature reserves.

## 2. Materials and Methods

### 2.1. Definition of Soil Diversity

In this study, soil diversity was quantified using both a soil map and a soil profile data set (described in the next section), which have been developed within the framework of the Genetic Soil Classification of China (GSCC) [14]. The GSCC contains six hierarchical levels including order, suborder, great group, subgroup, family, and species.  Table 1 shows the summary of the current number of taxa at different taxonomic levels of the GSCC.  Table 2 shows the distribution patterns and major distinguishing characteristics of the 11 soil orders.

**Figure 1:**
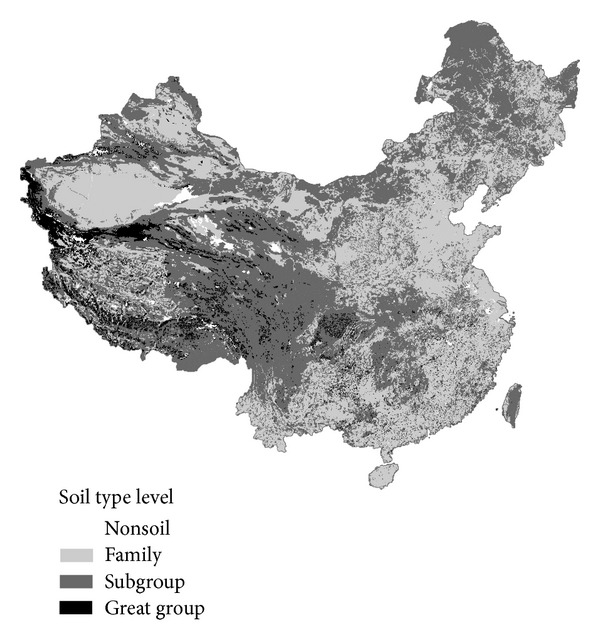
Soil taxonomic level of the 1 : 1,000,000 soil map of China [13].

We used two simple numerical measurements to quantify soil diversity [4]: (a) “taxon density”: number of taxa/area by region and (b) “taxon abundance”: total area of each soil class in a region. In the following sections, “soil classes” only include soil taxa at the great group, subgroup, and family levels if not specified otherwise. We defined rare and uncommon soils based on the number of taxa at a soil taxonomic level because the rarity of the soils depends on the soil taxonomic level (high level soil classes have fewer taxa than low level ones and occupy more area), so that the rarity of different taxonomic levels is comparable and it can be made better by the use of the taxonomic information of the soil map. Thus, the taxonomic level of the soil map (Figure 1) should be referenced when the soil diversity is analyzed.

The definitions of rare and uncommon soils are as follows: (a) rare soils: less than 200, 600, and 2,000 km^2^ at the family, subgroup, and great group levels, respectively; (b) unique soils: existing in only one province; (c) rare-unique soils: occurring in only one province, whose total area is less than 2,000, 6,000, and 20,000 km^2^ at the family, subgroup, and great group levels, respectively. Finally, for those natural soils (anthropogenic soils were excluded), we defined (d) endangered soils as (1) those rare or rare-unique soils that have lost more than 50% of their area due to various land disturbance by agriculture and construction, (2) those soils (regardless of whether they are rare) that have lost more than 50% of their area to land disturbance and that have less than 100, 300, and 1,000 km^2^ of undisturbed area at the family, subgroup, and great group levels, respectively, or (3) those unique soils that have lost more than 50% of their area to land disturbance and that have less than 1,000, 3,000, and 10,000 km^2^ of undisturbed area at the family, subgroup, and great group levels, respectively. The second and third types of endangered soils have similar undisturbed areas of the first type, which is a logical extension of the definition of Amundson et al. [4]. Soils with 90–100% of their total area disturbed are defined as extinct.

### 2.2. Data Acquisition and Analysis

**Figure 2:**
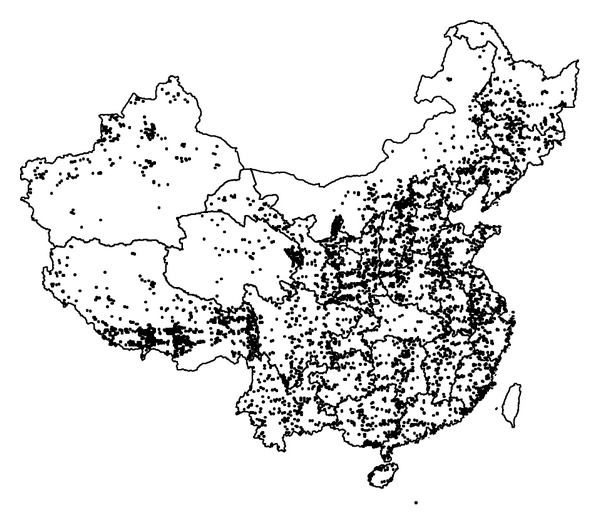
Location of soil profiles of China [15].

The two soil datasets that have been used in this study were the 1 : 1,000,000 soil map of China [13] (Figure 1) and the 8,979 soil profiles [15] (Figure 2) classified by GSCC, both of which were compiled from the Second National Soil Survey of China conducted in the 1980s. The soil map was based on field sampling (including the soil profiles used in this paper), remotely sensed data, and expert knowledge. This soil map was used to calculate the area of different soils. Each of the 925 soil map units in the soil map contains only one soil class, which are at the great group, subgroup, or family levels (Table 1 and Figure 1).The concept of soil map unit was demonstrated in our previous paper [16]. There are a number of soil polygons belonging to a soil map unit in the soil map. There are fewer classes at each soil taxonomic level in the soil map than the GSCC system, because some soils were not considered in the process of map generalization. The area of soil map polygons (overall 94303 polygons) was tabulated to obtain the total area of a soil class in China. There are 7822 different soil classes of soil profiles, and 7477 of them are at the soil species level (Table 1). However, we only used the soil taxonomic information at the family or higher taxonomic levels to assess soil diversity for the convenience of comparing with the soil map.

Soil disturbance in China was determined using the 1 km Grid China Land Use Data (GCLU) of 2005 [17]. To obtain the number and location of the endangered soils, the cultivated land and construction land layers were overlaid with the soil map. In the soil map, the rare and rare-unique soils were identified according to their definition. Then, we analyzed soil diversity by provinces and equal-area grids. Various grid sizes were tested, and finally the 100 km × 100 km grid size was chosen because it can show enough spatial details with a moderate number of grids. In addition, the 1 km GCLU data derived for late 1980s, 1995, and 2000 were also used in our analysis using the same procedure. As a result, the land-use effect on the soils over time could be assessed. The above spatial analyses were done using the ArcGIS software.

The endangered status of soil profiles was determined according to the results of the analysis based on the soil map. If a soil class on the soil map was endangered, the soil profiles belonging to this class were also considered endangered, or vice versa.

## 3. Results

### 3.1. Soil Diversity

**Figure 3:**
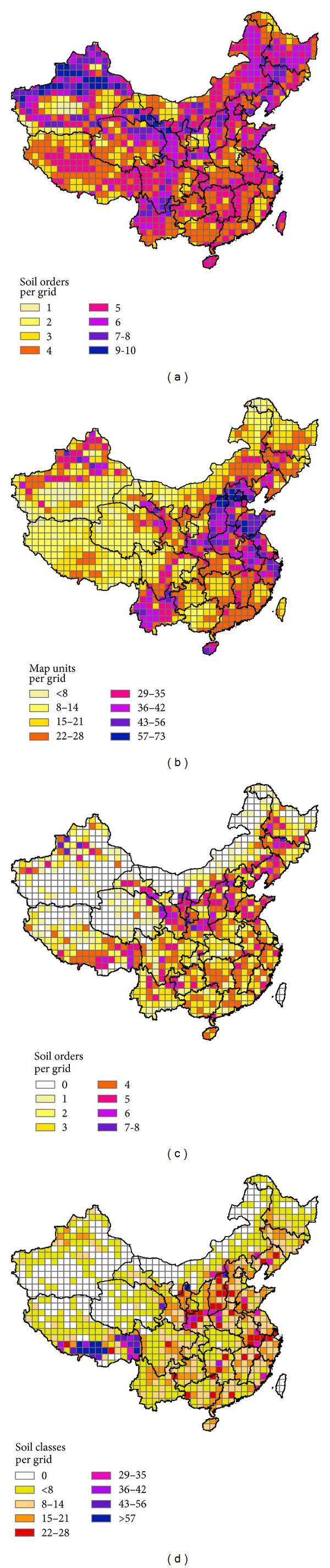
Soil orders (a) and soil map units at lower taxonomic levels (b) per grid in China based on soil map and soil orders (c) and soil classes at lower taxonomic levels (d) per grid in China based on soil profiles. Each grid is 100 km × 100 km in area. (The taxonomic level of the soil map is shown in Figure 1.)

Grids with a high quantity of soil orders were located in the northwest, northeast, and southwest of China (Figure 3(a)). The distributions of soil diversity at the soil suborder, great group, and subgroup levels had similar spatial patterns with those at the order level but they have been demonstrated to be less abundant in the northeast. The spatial pattern of the number of taxonomic classes at lower hierarchical levels per grid was quite different (Figure 3(b)). Grids with higher numbers of soil classes were located in the northwest, the north, the southern part of the southwest, and the northern part of the southeast. Areas with either low or high temperature or rainfall had low pedodiversity, as climate becomes the major limiting factor to soil formation [18]. The soil diversity at high levels (subgroup and above) was mainly a reflection of bioclimate (for zonal soils) and hydrologic (for intrazonal soils) factors. As the GSCC takes the local variation of soil-forming factors into account at the soil family level, soil diversity was mainly a reflection of the difference in parent material, topography, and hydrologic conditions. However, due to the incomplete information at the soil family level in the soil map of China some families were absent from the maps of soil diversity distribution, particularly in the south, northeast, and the northern part of southwest (Figure 1), where the presence of more soil families was expected due to the diversity of soil-forming factors in these areas.

The distributions of soil diversity based on the soil profiles were quite different from that based on the soil map (Figure 3). This was due to the distinct sampling density of the soil profiles in different areas. Most of the provinces had fewer soil orders based on the soil profiles than based on the soil map, which indicated that the taxonomic coverage of the soil profiles was not good at the soil order level. On the other hand, most provinces had more soil classes based on the soil profiles than those based on the soil map, particularly Tibet, which had a much more detailed soil profile database. As a result, the current soil map did not represent all the soil classes in the soil profiles and there should be more soil classes. Due to the above analysis, the actual soil diversity in China is expected to be much higher than the diversity shown by the available data.

Soil diversity was also analyzed by province (Table 3). Gansu, Inner Mongolia, Qinghai, Shanxi, and Xinjiang had soils belonging to 11 different orders. The first four provinces are located in the transition zone of the semihumid to semiarid and arid climates, while Xinjiang has a wide range and vertical soil zones on the mountains. With the exception of Ferralsols, all of the remaining soil orders existed in these provinces because of their diversified soil-forming conditions. On the contrary, the diversity of the soil orders was not high in the tropic and subtropical provinces, where only one kind of zonal soils (i.e., Ferralsols) existed. In terms of the number of soil map units at the great group, subgroup, or family levels, Hebei had the greatest number (235), followed by Xinjiang (218), Inner Mongolia (207), and Henan (168). For the number of soil taxa per 10,000 km^2^, Hainan had the highest density (21.79), followed by Jiangsu (13.9), Ningxia (12.76), and Taiwan (12.32), while Tibet (0.88) and Xinjiang (1.33) had the lowest.

### 3.2. Land Use and Soil Diversity

There are 231 anthropogenic soils on the soil map of China, which occupy approximately 12% of the nation's total soil area. These anthropogenic soils developed under long periods of cultivation [19], and their properties are quite different from their natural counterparts. As anthropogenic soils have already replaced the original natural soils, they were excluded when assessing land-use effects in the recent 3 decades on soil diversity.

**Figure 4:**
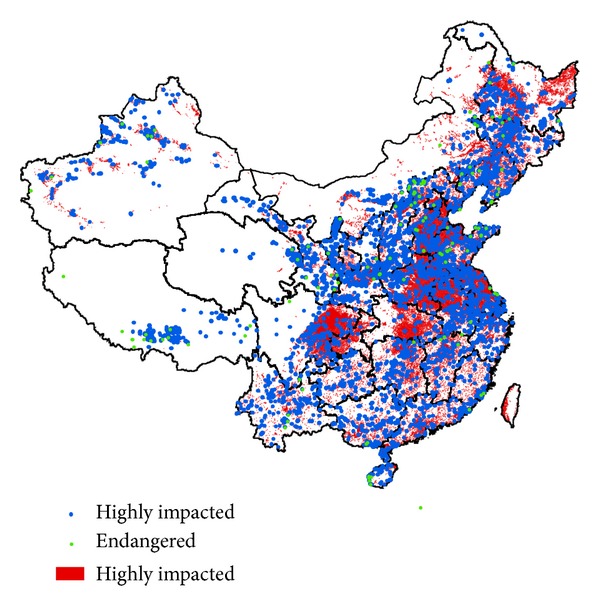
Soils that have 50% or more of their area impacted by construction and cultivation (the taxonomic level of the soil map is shown in Figure 1).

Two types of land-use effects (i.e., construction and cultivation) were assessed in this study. Soils become exposed to big changes due to the intensive human disturbance. They are not likely to remain the same as their natural counterparts. Mankind can be as a soil forming factor, and it may create new soils such as anthropogenic soils. However, the original natural soils do not exist anymore even if the pedodiversity increased, and this may cause threats to some soils or even lead to soil extinction. Although construction land occupies only 0.18 million km^2^ (approximately 1.98%), rapid urbanization in China was considered a great threat to soil protection and food security [12].  Figure 4 shows 359 soils in the soil map which had 50% or more of their area impacted by cultivation and construction, regardless of their total extent. Overall, 52% of the profiles are highly impacted by construction or cultivation.

At the soil order and great group levels, certain soils are more heavily affected by construction and cultivation than others as a result of uneven development of construction and cultivation activities (Table 4). All of the anthropogenic soils had high percentages of impacted area (approximately 60% or more). With the exception of Anthrosols, the four soil orders with the greatest areas of disturbance were: semiaqueous soils (62%), semi-Alfisols (45%), Pedocal (24%), and Ferralsols (20%). For natural soils at the great group level, the impact of land use was not equal. Black soils, which are distributed in the plains of northeast China, were most heavily disturbed (80%) due to their high content of organic matter, which benefits crop growth. Other soils that were highly devoted to agricultural land include yellow-cinnamon soils (67%), albic soils (52%), dark loessial soils (49%), purplish soils (45%), castano-cinnamon soils (44%), red clay soils (44%), and cinnamon soils (42%). With the exception of cold brown calcic soils, all of the soils in the Alpine soil order were nearly undisturbed. Although amorphic soils are immature soils, most great groups of this order were relatively heavily disturbed (over 40% of their area).

### 3.3. Unique, Rare, and Rare-Unique Soils

“Soil endemism” refers to soils occupying very small areas in a geographical distribution [20]. Most soils existed in five or fewer provinces (approximately 81% of the total), and only 71 soils appeared in more than ten provinces. 302 soils were identified as unique soils in China. Xinjiang has the greatest number of unique soils (75), followed by Tibet (30), Hebei (29), Qinghai (22), and Yunnan (22).

**Figure 5:**
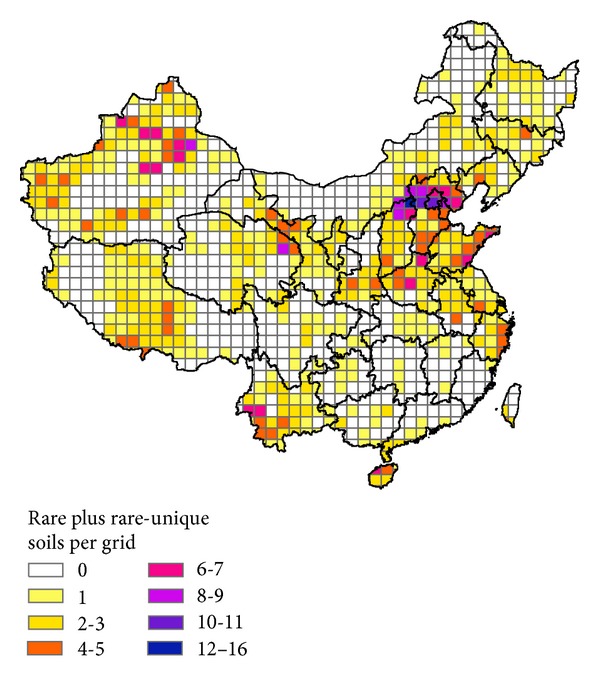
Rare plus rare-unique soils per grid in China (the taxonomic level of the soil map is shown in Figure 1).

We found 332 rare or rare-unique soils, occupying 1.3% of China's land area.  Figure 5 shows that the rare or rare-unique soils were distributed mainly in the north, east, southwest, and northwest of China. Table 4 shows the number of rare or rare-unique soils in each province. Xinjiang had the greatest number of rare or rare-unique soils (64), followed by Hebei (54), Shanxi (27), Qinghai (26), and Tibet (24). In terms of rare or rare-unique soil density, Hainan led the nation (3.6 soil cs per 10,000 km^2^), followed by Hebei (2.5), Shanxi (1.7), Jiangsu (1.6), and Zhejiang (1.6).

### 3.4. Endangered Soils

**Figure 6:**
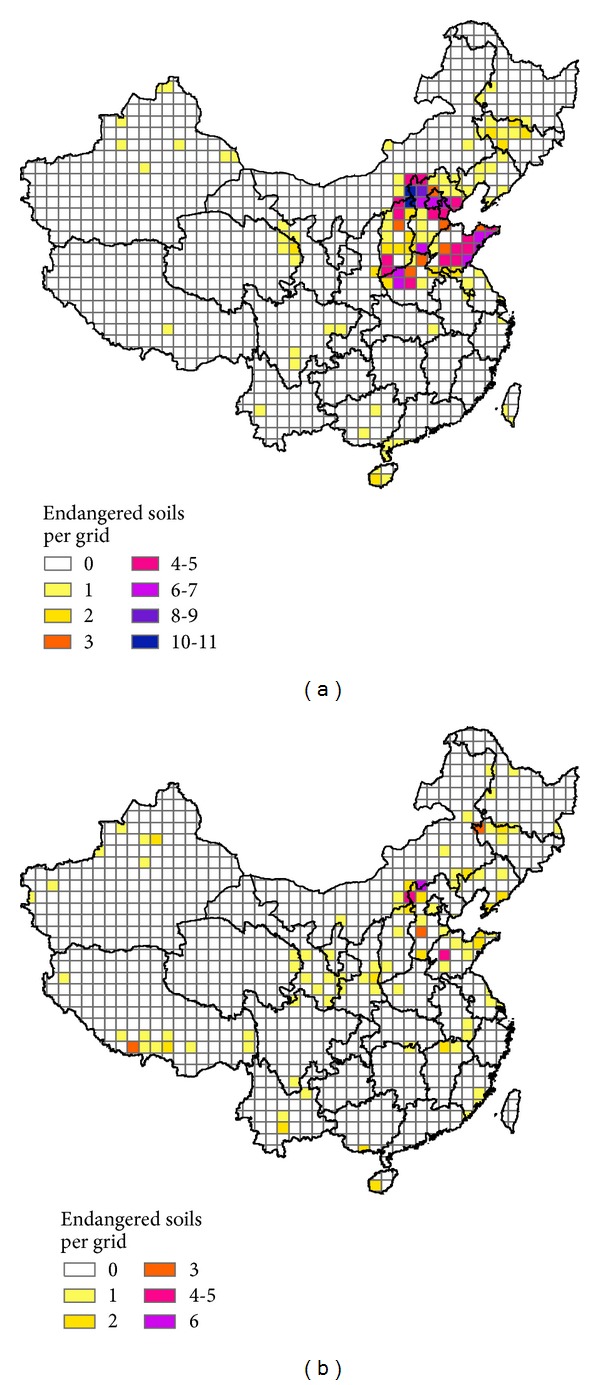
Endangered soils per grid based on soil map (a) and based on soil profiles (b) in China (the taxonomic level of the soil map is shown in Figure 1).

A total of 88 endangered soils with a total area of 19.2 thousand km^2^ were found in China, occupying approximately 0.2% of China's land area (Table 4, Figure 6(a)). Most endangered soils were located in the north of China. Hebei had the greatest number of endangered soils (37), followed by Shanxi (19), Shandong (14), and Henan (11). With respect to endangered soil density, these provinces also led the nation. These provinces are adjacent and located in the North China Plain and the Loess Plateau. Although Xinjiang, Qinghai, and Tibet had large numbers of rare or rare-unique soils, the number of endangered soils in these provinces was small because land-use activities are less intensive. The endangered soils belonged to 10 soil orders and 19 soil great groups (Table 4). More than half of the endangered soils were in the Semi-Alfisols (24) and Pedocal (23) orders. Cinnamon soils had the greatest number of endangered soils (22), followed by castanozems (12), castano-cinnamon soils (8), bog soils (8), and meadow soils (7). In China, 17 soils might be considered “extinct” (90–100% land conversion) (Table 3), which were located in the intensively disturbed area of the north (Shanxi, Henan, Anhui, and Shandong). Most of the conversion was caused by cultivation. Only 6 soils had more than 20% of their area converted by construction land use. Four provinces had a ratio of endangered soils to rare soils that was greater than 0.5, that is, Shandong (0.74), Shanxi (0.70), Hebei (0.69), and Jilin (0.50). High ratios of endangered soils to rare soils indicate intensive land disturbance. It is even worse in these places, as their endemic soils (more importantly if they are China's endemic soils) are under pressure. According to the Harmonized World Soil Database [21], 17 soils were unique in China (but not endangered), which include Gelic Leptosols (14), Albic Lixisols (1), Fimic Anthrosols (1), and Gypsic Solonetz (1), based on FAO-90 (Food and Agriculture Organization of the United Nations) soil classification [22]. However, the FAO-90 classification, which has only 155 soil units, is not detailed enough to determine all of the soil endemism.

Overall 133 soil profiles belonged to the endangered soils, accounting for 1.5% of the total number of soil profiles (Figures 4 and 6(b)). Approximately one quarter of the endangered soil profiles were not occupied by cultivation or construction yet, particularly for those on the Tibet Plateau. The distributions of endangered soil profiles and soil map units were quite different (Figure 6). This is partly because the sampling of profiles is based mainly on the availability of legacy data and not on an area-weighted method, and partly because the soil map has missed some of the soils. The analysis of the soil profiles can offer some complementary information on endangered soils.

The soil map of China was compiled based on a survey from 1979 to 1994 and almost all of the field survey took place during the 1980s, and it reflects the state of soils in the 1980s. During the past 30 years, the stress on the soil has changed over time (Table 5). The number of endangered soils increased, while the number of extinct soils seemed to be stable. 94 soils were identified as endangered during this period. However, only 70% of endangered soils were always disturbed by cultivation and construction. Other endangered soils were either newly severely occupied by construction and cultivation or occupied at once but were later changed into other land categories, such as forest and grassland. The soils that were once disturbed were not likely to resume, as the current land use now may not likely be the same before the soils were disturbed.

### 3.5. The Protection Status of Soils

**Figure 7:**
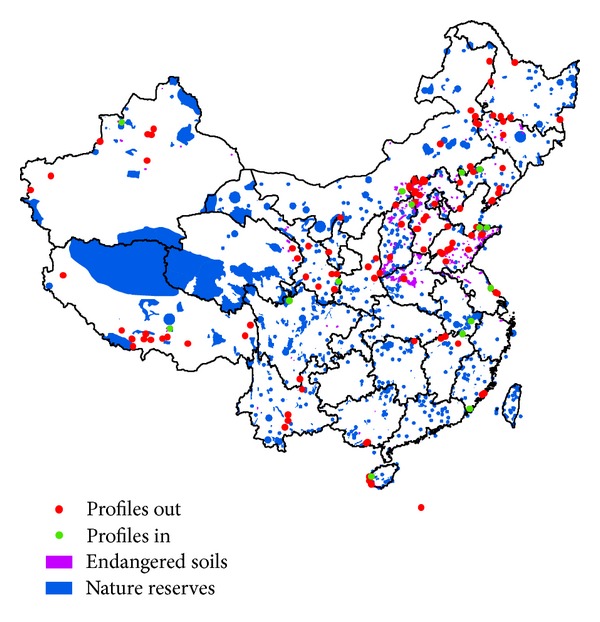
Endangered soils and nature reserves. The symbol of endangered soils is emphasized. “Profiles in” are endangered soil profiles in the nature reserves, and “profiles out” are endangered soil profiles out of the nature reserves.

Although the planning of ecological functions has taken soil erosion and desertification into account [23], soil diversity has not been considered as a priority in soil conservation practices in countries around the world. In China, many endangered soils (84% in area and 89% in number of soil profiles) are outside nature reserves (Figure 7). Protection of such soils should be a high priority in the creation of future nature reserves.

## 4. Discussion

In the past 300 years, as population growth led to increasing amount of agriculture and construction land use in China, human impacts on the natural environment have become more intensive and have been expanding [24]. Although agricultural soils have been highly treasured by farmers in China, there is a lack of recognition of soil diversity and why natural soils as a whole, or specific natural soils individually, are important to the society [4]. For the first time, through this study, a clear knowledge on the rare and threatened soils in China is obtained. Our findings will have significant implications for future soil protection planning in China.

The soil extinction seemed to have stopped in southern China (Figure 6) but was prevalent in northern China. However, the extinction may be underestimated in Hunan and Hubei due to the lack of taxonomic information at the family level. Anthropogenic soils, which have replaced their natural soil counterparts under the GSCC classification system, may imply that some soils have already been extinct. In this sense, “extinction” had already happened in the most populated regions of China. However, the GSCC does not reflect all of the aspects of human impacts on soils, even for some significant changes in soil properties. For example, the black soil in northeast China has been cultivated intensively since 1949 when the People's Republic of China was funded, and the organic matter in these black soils has decreased dramatically accompanied by noticeable soil erosion. However, none of the four map units of black soil in the soil map was considered endangered soils due to the effects of land use.

The list of endangered soils in this study was identified by considering only the rarity and the land disturbance, but the importance of soils in their economic, ecosystem, scientific, and historical/cultural value is quite different [25] and should be studied in the future. From a purely economic perspective, the endangered soils in the amorphic soils, alkaline-saline soils, desert soils, and Aridisols orders have little economic value and thus may not need to be preserved. However, the so-called “precautionary principle” [26] in habitat conservation planning requires that the diversity of natural soils be maintained because we lack the scientific understanding of their full values and functions [4].

Different soils, with their unique physical and chemical properties and biological functions, are related to the diversity of soil biota, which is an uprising field of scientific research [27]. Almost all of the processes in the soils are related to soil biota, which has the greatest number of species in the terrestrial ecosystem. Pedodiversity was found to be strongly correlated to biodiversity at the global level [28]. Many threatened and endangered plants have specific soil property requirements, which can be used to predict rare plant habitats [29]. The loss of a soil may change the whole soil ecosystem and cause a loss of its corresponding soil organisms [30]. Overall, the loss of some soils represents a substantial loss of biodiversity below ground and above ground in the corresponding biological communities [31].

The inherent uncertainty brought by the soil data is greatly outweighed by the insights that the results provide, and it is very likely that the assessment of endangered soils in our study is an underestimate for several reasons as discussed elsewhere [4]. The soil map of China that was used in this study does not give information at the soil species level; therefore the analysis omitted many endangered soils. Furthermore, parts of the soil map were mapped at the soil subgroup or great group level. This may be an important reason for the few endangered soils that were found at coarsely mapped areas, particularly in south China in this study. Each map unit in the soil map of China has only one soil class, and the purity of the soil map of China is less than 50% to 65% [32], which means that other soil classes in a map unit were not presented in our analysis. In addition, the soils that were disturbed once but not persistently are actually gone, as their nature has already been altered (Table 5). We do not have precise land-use data before the 1980s nor do we have high temporal frequency land-use data to fully assess land-use stress on the soils. All of the above factors combined increase the level of underestimation of the land-use effects on soil diversity in China.

Because of soil endemism [20], pedodiversity conservation should be considered an important aspect in international cooperation. If a unique soil in a country disappears, it will be a loss of soil diversity for the whole world. It is difficult to determine China's endemic soils due to the lack of a detailed soil map of the world using the same classification system [16]. The World Reference Base for Soil Resources (WRB) [33] provides opportunities to compare soils worldwide, although a truly universal classification system does not yet exist [34]. At the working scale included in this study, it is suggested to use enough qualifiers (4) attached to the main groups that can provide enough details to differentiate soils. This will require a large amount of resources and well-coordinated international collaboration [35]. In addition, a more comprehensive soil survey is needed in order to have a clearer picture of the soil diversity at a finer level of soil classification.

The loss of soil diversity caused by land-use change and other changes, such as climate change, desertification, and soil pollution, is not fully accounted for in this study. For example, irrational and intensive land use on a fragile Karst geoecological environment is causing serious soil erosion and rocky desertification in southwest of China [36]; urban and agriculture soils are suffering from heavy metal pollution [37, 38], and global warming is thawing frozen soils on the Tibetan Plateau [39].

## 5. Conclusions

Although there were some limitations due to the lack of detailed data, this study was the first attempt to give some insights about the effects of land use on pedodiversity at the national scale. First, agriculture and construction land use have significant influence on pedodiversity. Second, the distribution of endangered soils was uneven across the country. Third, most of the endangered soils remain unprotected out of natural reserves. More attention should be paid to soil diversity in conservation activities.

## Figures and Tables

**Table 1:** Number of taxa or map units at taxonomic levels in China.

Taxonomic level	Number of taxa in GSCC^a^	Number of map units^b^	Number of taxa in soil profiles^c^
Order	12	—	—
Suborder	29	—	—
Great group	61	22	17
Subgroup	231	215	129
Family	909	688	199
Specie	—	—	7477

^a^GSCC, Genetic Soil Classification of China.

^b^Map units in the 1 : 1,000,000 soil map of China.

^c^This is according to the most detailed taxonomic information of a soil profile.

**Table 2:** Distribution and brief description of characteristics of the soil orders in China.

Order	Distribution	Characteristics
Alfisols	Humid region	Calcium carbonate leached well, acid or neutral, clay-enriched B horizons
Semi-Alfisols	Semihumid region	Weak leaching, neutral to slight alkaline, calcium carbonate illuviated, argillation of different degree
Pedocals	Semiarid and arid regions	Horizons with off-white lime
Aridisols	Arid region	Arid A horizons and any other subhorizons
Desert soils	Most arid region	Hydromica as major clay mineral, crust with vesicles and platy horizon, horizon with rich gypsum and salt
Amorphic soils	Azonal	Week pedogenesis, characteristics of parent material
Semiaqueous soils	Intrazonal	Groundwater invasion or temporarily stagnant water, soil humification surface horizon and rust horizon of oxidation-reduction
Aqueous soils	Intrazonal	Surface water or groundwater near the surface, crude humification or peat surface layer, gley horizon
Alkaline-saline soils	Intrazonal	Soil property and profile change caused by soil salt or alkalization, no crops
Anthrosols	Nonzonal	Characters caused by long period of cultivation
Alpine soils	Plateau and alpine area	Week humification, freezing-thawing morphology, low soil depth, coarse soil texture, low mineral chemical decomposition
Ferralsols	Warm-wet climate zone	Desilication, Fe and Al enriched, bioaccumulation

**(a):** 

Province^a^	Number					Ratio of endangered soil to rare soil^d^	Number/10,000 km^2^		
	Order	Map units^b^	Rare plus rare-unique	Endangered	Extinct^c^		Map units	Rare plus rare-unique	Endangered
Anhui	8	130	12	2	1	0.17	9.28	0.86	0.14
Fujian	6	48	1	0	0	0.00	3.96	0.08	0.00
Gansu	11	157	10	1	0	0.10	3.88	0.25	0.02
Guang-dong	6	68	3	1	0	0.33	3.83	0.17	0.06
Guangxi	6	75	10	2	1	0.20	3.17	0.42	0.08
Guizhou	5	51	1	0	0	0.00	2.90	0.06	0.00
Hainan	6	73	12	3	0	0.25	21.79	3.58	0.90
Hebei	8	235	54	37	6	0.69	10.92	2.51	2.04
Henan	8	168	23	11	5	0.48	10.14	1.39	0.66
Heilong-jiang	9	76	11	2	1	0.18	1.68	0.24	0.04
Hubei	6	96	5	1	0	0.20	5.17	0.27	0.05
Hunan	6	58	1	0	0	0.00	2.74	0.05	0.00
Jilin	9	92	12	6	1	0.50	4.82	0.63	0.31
Jiangsu	8	149	17	3	2	0.18	13.90	1.59	0.28
Jiangxi	5	67	4	0	0	0.00	4.01	0.24	0.00
Liaoning	8	101	9	3	0	0.33	6.97	0.62	0.21
Inner Mongolia	11	207	20	9	0	0.45	1.80	0.17	0.08
Ningxia	10	66	6	0	0	0.00	12.76	1.16	0.00
Qinghai	11	130	26	2	0	0.08	1.81	0.36	0.03
Shandong	7	148	19	14	3	0.74	9.59	1.23	0.91
Shanxi	9	149	27	19	3	0.70	9.51	1.72	1.21
Shaanxi	11	133	11	0	0	0.00	6.46	0.53	0.00
Sichuan	8	114	10	2	0	0.20	2.35	0.21	0.04
Taiwan	6	44	4	1	0	0.25	12.32	1.12	0.28
Tibet	9	106	24	1	0	0.04	0.88	0.20	0.01
Xinjiang	11	218	64	3	0	0.05	1.33	0.39	0.02
Yunnan	9	148	22	1	0	0.05	3.87	0.57	0.03
Zhejiang	7	117	16	3	1	0.19	11.37	1.56	0.29
Chong-qing	5	43	1	0	0	0.00	5.21	0.12	0.00

^a^Some small administration districts were merged into adjacent provinces. Hong Kong and Macao were merged into Guangdong, Beijing and Tianjin were merged into Hebei, and Shanghai was merged into Jiangsu.

^b^The map units are at the great group, subgroup, or family levels.

^c^The endangered soils in China and the percentage of their area that has been disturbed by cultivation and construction are given in Table 3(b).

^d^Number of endangered soils can be bigger than the rare plus rare-unique soils because some endangered soils are not rare plus rare-unique soils.

**(b):** 

Percent of disturbed area	Cultivation/construction^a^	Construction (>20)^b^
50–60	21	1
60–70	19	0
70–80	13	0
80–90	18	1
90–100	17	4

Total	88	6

^a^Number of endangered soils in China with percentage of land (as defined in column 1) devoted to combined cultivation and construction use.

^b^Number of endangered soils in China with more than 20% land devoted to construction use.

**Table 4:** Percentage of soil order and great group affected by development in China.

Order	%			En^c^	Great group	%			En^c^
	Con^a^	Cul^b^	Total			Con^a^	Cul^b^	Total	
Alfisols	1.49	18.21	19.70	6	Brown coniferous forest soils	0.04	0.21	0.25	
					Brown earths	0.00	0.00	0.00	
					Yellow-brown earths	0.37	16.82	17.20	
					Yellow-cinnamon soils	8.84	67.10	75.94	
					Brown earths	2.86	22.59	25.45	6
					Dark-brown earths	0.43	10.15	10.58	
					Albic soils	2.74	51.74	54.48	

Semi-Alfisols	4.94	39.62	44.57	24	Torrid red soils	1.70	28.97	30.67	2
					Cinnamon soils	6.43	42.49	48.92	22
					Gray-cinnamon soils	0.79	9.91	10.70	
					Black soils	5.37	74.76	80.12	
					Gray forest soils	0.18	4.66	4.84	

Pedocal	1.67	22.79	24.45	23	Chernozems	2.34	33.42	35.75	3
					Castanozems	1.21	15.28	16.49	12
					Castano-cinnamon soils	2.85	43.56	46.42	8
					Dark loessial soils	3.25	48.85	52.11	

Aridisols	0.58	6.69	7.27	1	Brown Pedocals	0.38	3.12	3.50	
					Sierozems	1.73	26.68	28.41	1

Desert soils	0.30	2.00	2.30	1	Gray desert soils	1.22	10.46	11.69	1
					Gray-brown desert soils	0.18	0.66	0.84	
					Brown desert soils	0.18	1.27	1.45	

Amorphic soils	0.81	18.51	19.32	9	Cultivated loessial soils	1.44	41.22	42.66	
					Red clay soils	3.59	44.42	48.01	
					Alluvial soils	4.67	38.46	43.14	5
					Takyr	0.50	2.48	2.98	
					Aeolian soils	0.31	3.75	4.06	1
					Limestone soils	0.71	24.28	25.00	
					Volcanic soils	3.31	35.29	38.60	
					Purplish soils	0.87	45.23	46.10	
					Litho soils	0.30	2.96	3.26	1
					Skeletol soils	1.21	18.45	19.66	2

Semi-Aqueous soils	8.31	53.54	61.85	7	Meadow soils	3.14	34.86	38.01	7
					Lime concretion black soils	14.03	83.97	97.99	
					Mountain meadow soils	0.15	4.25	4.40	
					Shrubby meadow soils	0.34	7.54	7.89	
					Fluvo-aquic soils	13.00	70.41	83.41	

Aqueous soils	0.72	12.20	12.92	8	Bog soils	0.72	12.21	12.94	8
					Peat soils	0.60	11.93	12.54	

Alkaline-saline soils	2.98	12.52	15.50	6	Saline soils	1.15	12.98	14.13	1
					Desert solonchaks	0.12	2.65	2.77	
					Coastal solonchaks	25.42	32.51	57.93	5
					Sulphate soils	13.09	13.55	26.64	
					Frigid plateau solonchaks	0.00	0.00	0.00	
					Solonetzs	1.90	26.50	28.40	

Anthrosols	7.86	59.70	67.56		Paddy soils	7.91	59.51	67.42	
					Cumulated irrigated soils	7.82	63.91	71.74	
					Irrigated desert soils	5.92	59.68	65.59	

Alpine soils	0.02	0.27	0.28		Felty soils	0.01	0.06	0.07	
					Dark felty soils	0.05	0.95	1.01	
					Frigid calcic soils	0.00	0.01	0.01	
					Cold calcic soils	0.04	0.79	0.84	
					Cold brown calcic soils	1.01	14.65	15.66	
					Frigid desert soils	0.00	0.00	0.00	
					Cold desert soils	0.00	0.00	0.00	
					Frigid frozen soils	0.00	0.01	0.02	

Ferralsols	1.20	19.01	20.22	3	Humid-thermo ferralitic	3.51	29.58	33.10	1
					Latosolic red earths	2.48	19.61	22.09	1
					Red earths	1.00	17.78	18.78	1
					Yellow earths	0.26	19.89	20.14	

^a^Land for construction.

^b^Land for cultivation.

^c^Number of endangered soils.

**Table 5:** Land use stress on soil over time.

Number	1980s^a^	1995	2000	2005	Any^b^	Persistent^c^
Endangered soils	78	72	80	88	94	66
Extinct soils	16	14	17	17	23	10

^a^Late 1980s.

^b^Soils disturbed in any years.

^c^Soils disturbed all the time.
